# Inhibition of Macrophage Migration Inhibitory Factor Ameliorates Ocular *Pseudomonas aeruginosa*-Induced Keratitis

**DOI:** 10.1371/journal.ppat.1000826

**Published:** 2010-03-26

**Authors:** Mihaela Gadjeva, Jill Nagashima, Tanweer Zaidi, Robert A. Mitchell, Gerald B. Pier

**Affiliations:** 1 Department of Medicine, Channing Laboratory, Brigham and Women's Hospital, Harvard Medical School, Boston, Massachusetts, United States of America; 2 James Graham Brown Cancer Center, University of Louisville, Louisville, Kentucky, United States of America; Harvard Medical School, United States of America

## Abstract

*Pseudomonas aeruginosa* causes severe sight-threatening corneal infections, with the inflammatory response to the pathogen being the major factor resulting in damage to the cornea that leads to loss of visual acuity. We found that mice deficient for macrophage migration inhibitory factor (MIF), a key regulator of inflammation, had significantly reduced consequences from acute *P. aeruginosa* keratitis. This improvement in the outcome was manifested as improved bacterial clearance, decreased neutrophil infiltration, and decreased inflammatory responses when *P. aeruginosa*-infected MIF knock out (KO) mice were compared to infected wild-type mice. Recombinant MIF applied to infected corneas restored the susceptibility of MIF deficient mice to *P. aeruginosa*-induced disease, demonstrating that MIF is necessary and sufficient to cause significant pathology at this immune privileged site. A MIF inhibitor administered during *P. aeruginosa*-induced infection ameliorated the disease-associated pathology. MIF regulated epithelial cell responses to infection by enhancing synthesis of proinflammatory mediators in response to *P. aeruginosa* infection and by promoting bacterial invasion of corneal epithelial cells, a correlate of virulence in the keratitis model. Our results uncover a host factor that elevates inflammation and propagates bacterial cellular invasion, and further suggest that inhibition of MIF during infection may have a beneficial therapeutic effect.

## Author Summary

*Pseudomonas aeruginosa* can induce infections that lead to a rapid loss of visual function. The current therapy includes antibiotic treatment which reduces the bacterial burden; nevertheless, tissue damage occurs as a result of an uncontrolled inflammation. Therefore, new therapeutic approaches are needed that will combine antimicrobial treatment with anti-inflammatory treatment. Our experiments have uncovered that mice deficient for macrophage migration inhibitory factor (MIF) recovered from acute bacterial infection more efficiently than wild-type control mice. This improvement was manifested as improved bacterial clearance, and decreased inflammatory responses in the MIF knockouts when compared to *P. aeruginosa-*infected wild-type control mice. We find that treatment of the infected mice with small molecule inhibitor of MIF activity after the onset of the infection promoted recovery from disease. This approach will facilitate generation of novel treatment strategies for bacterial keratitis.

## Introduction

Eye trauma and contact lens wear are the main factors that predispose to the development of infectious keratitis associated with vision loss and blindness [1],[2]. The organism most often isolated from contact lens associated corneal ulcers is *Pseudomonas aeruginosa* [3],[4]. Existing therapies often fail to control the excessive tissue damage that is induced during *P. aeruginosa* infection [5]. While antibiotic treatment reduces the bacterial burden, tissue damage still occurs as a result of an poorly-controlled local inflammation. Hence, new therapeutic modalities are needed to control the inflammatory response in addition to the antibiotic treatments. We hypothesized that the innate immunity factor–MIF (NP 002406)–could promote the pathogenic consequences of *P. aeruginosa* infection by potentiating local inflammation, and, if so, could be a suitable drug target for treatment.

MIF is an innate immunity molecule with ubiquitous tissue expression leading to induction of proinflammatory activities. MIF was originally described as a regulator of macrophage responses [6]. It directly or indirectly promotes expression of a large panel of pro-inflammatory cytokines including TNF-α (P01375), IFN-γ (P 01579), IL-1β (NP 000566), IL-2 (AAA 59140), IL-6 (CAG 29292), IL-8 (CAG 46948), MIP-2 (AAF 78449), NO, COX2 (P 00403), products of the arachidonic acid pathway and matrix metalloproteinases [7]. Interestingly, low levels of MIF can override the anti-inflammatory properties of glucocorticoids by reversing the inhibitory effect of glucocorticoid on production of TNF-α, IL-1β, IL-6 and IL-8 [8]. Detailed studies performed in the rat have shown that preformed MIF protein is released into the circulation within 6 hrs of LPS injection [9]. LPS toxicity is exacerbated by co-injection of recombinant MIF (rMIF) with LPS, whereas neutralization of MIF activity reduces the circulating levels of TNF-α by 50% and rescues mice from lethal LPS-induced endotoxic shock [10],[11]. All these properties of the MIF molecule suggest that MIF has a prominent regulatory role related to inflammation, likely with a critical function as an effector molecule that is active early in the course of infection with a pathologic function when continued production exacerbates inflammation, giving rise to attendant tissue pathology.

The contribution of MIF during responses to infections by a variety of pathogens, including bacteria, viruses, and parasites is currently an area of active research. Recent clinical correlative studies have demonstrated increased MIF levels and elevated MIF-dependent proinflammatory cytokines are produced during H1N5 influenza infection, dengue fever, and bacterial urinary tract infections [12],[13],[14]. These results demonstrate an important contribution of MIF to the pathogenesis of viral or bacterial induced inflammation and suggest a possible beneficial role of neutralizing MIF as an adjunctive therapeutic approach to treat the severe forms of disease.

In the eye, the high levels of MIF protein expression and consequent inhibition of cellular migration has supported the conclusion that MIF contributes to the establishment of the eye's immune privilege status due to immunosuppressive activities [15],[16],[17],[18]. While this could be important in the resting state, the strong impact MIF has on induction of inflammation in response to infection suggests a different role for this molecule during active infection. In this study, we tested whether inhibition of MIF in the eye led to a decrease in *P. aeruginosa*-induced pathology, and if this was associated with decreased PMN infiltration and thus decreased inflammation and corneal pathology. Overall, we found that inhibition of MIF had a pronounced salutary effect on eye pathology emanating from *P. aeruginosa* keratitis, which we associated with a better ability of PMN from MIF-deficient mice to mediate opsonic killing of this organism, making MIF a promising therapeutic target to control local inflammation in the context of *P. aeruginosa* corneal infection.

## Results

### Bacterial burdens after *Pseudomonas aeruginosa* eye infection are elevated in WT mice compared with MIF KO mice

**Figure 1:**
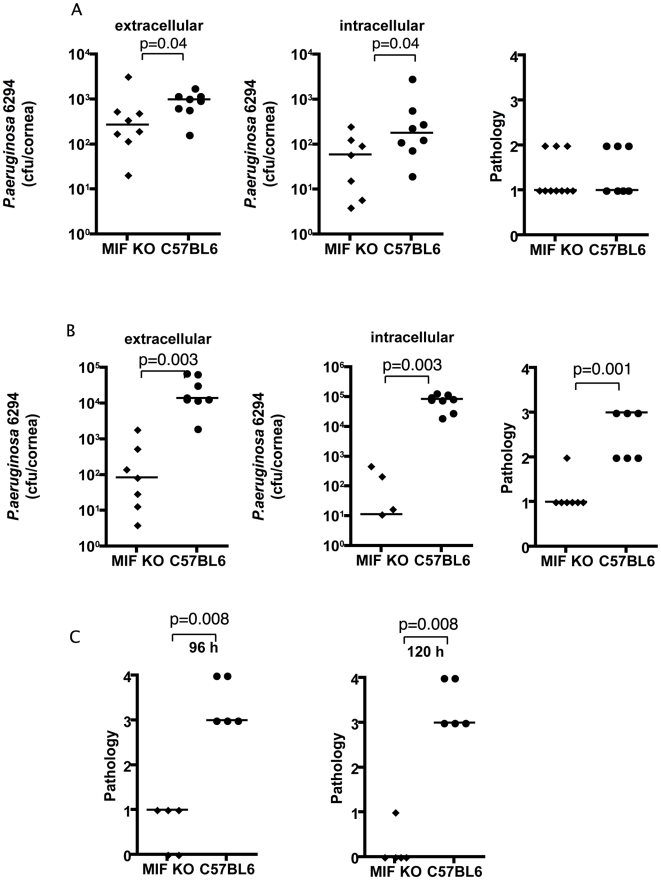
MIF KO mice are protected from *P. aeruginosa*-induced infection. **A.** Extracellular (left) and intracellular (middle) levels of *P. aeruginosa* strain 6294 and associated pathology scores (right) determined 24 h following infection with 10^6^ cfu onto eyes of MIF KO or WT C57Bl6 mice. Points indicate values for individual mice, bars the medians. Data are from a representative experiment out of two performed. **B.** Extracellular (left) and intracellular (middle) cfu of *P. aeruginosa* strain 6294 at 48 h following infection. Significantly different levels of 6294 were detected both extracellularly (left) (P = 0.0003), intracellularly (middle) (P = 0.0003) as were differences in pathology scores (right; P = 0.001). P values by Mann-Whitney U test. The data are from one representative experiment out of three performed. **C.** Significantly higher corneal pathology scores induced by *P. aeruginosa* at 96 h (P = 0.007) and 120 h (P = 0.009) after challenge. Points indicate individual mice, bars the medians. Data are from a representative experiment out of two performed.

To determine the effect of MIF on *P. aeruginosa*-induced keratitis, MIF KO and C57Bl6 WT mice were infected with *P. aeruginosa* strain 6294 and the bacterial levels in the corneas of mice measured to monitor disease progression. At 24 h after onset of infection there were modest but significantly (P = 0.04) lower bacterial levels in both the extracellular and intracellular samples from the corneas of the infected MIF KO mice when compared to C57Bl6 control animals (Fig. 1A). However, the corneal pathology scores of MIF KO and C57Bl6 were comparable at 24 h.

At 48 h after infection the differences between the MIF KO and C57Bl6 mice increased dramatically: there were about 100-fold fewer bacteria recovered from the corneal cell exterior in the infected MIF KO mice compared to the C57Bl6 controls. Even more dramatically, about 10,000 less intracellular bacteria were recovered from MIF KO mice when compared to C57Bl6 mice (Fig. 1B). Consistent with the reduced bacterial levels, the MIF KO mice infected with *P. aeruginosa* strain 6294 had a significant decrease in corneal pathology (P = 0.001).

As is known from published data [19], if a *P. aeruginosa* corneal infection is left to proceed for a longer period of time in C57Bl6 mice, perforation of the cornea occurs. This outcome was observed in an additional group of C57BL6 control mice, but not in MIF KO mice in the C57Bk6 background (Fig. 1C). This reduced pathology in the MIF KO mice was observed across a range of bacterial challenge doses from 1×10^6^ (data not shown) to 1×10^7^ cfu/mouse eye (Fig. 1C). The improved outcomes in MIF KO mice was also obtained with *P. aeruginosa* strain PAO1 48 h following infection (Figure S1).

### MIF deficiency regulates inflammatory responses during *P. aeruginosa* keratitis

**Figure 2:**
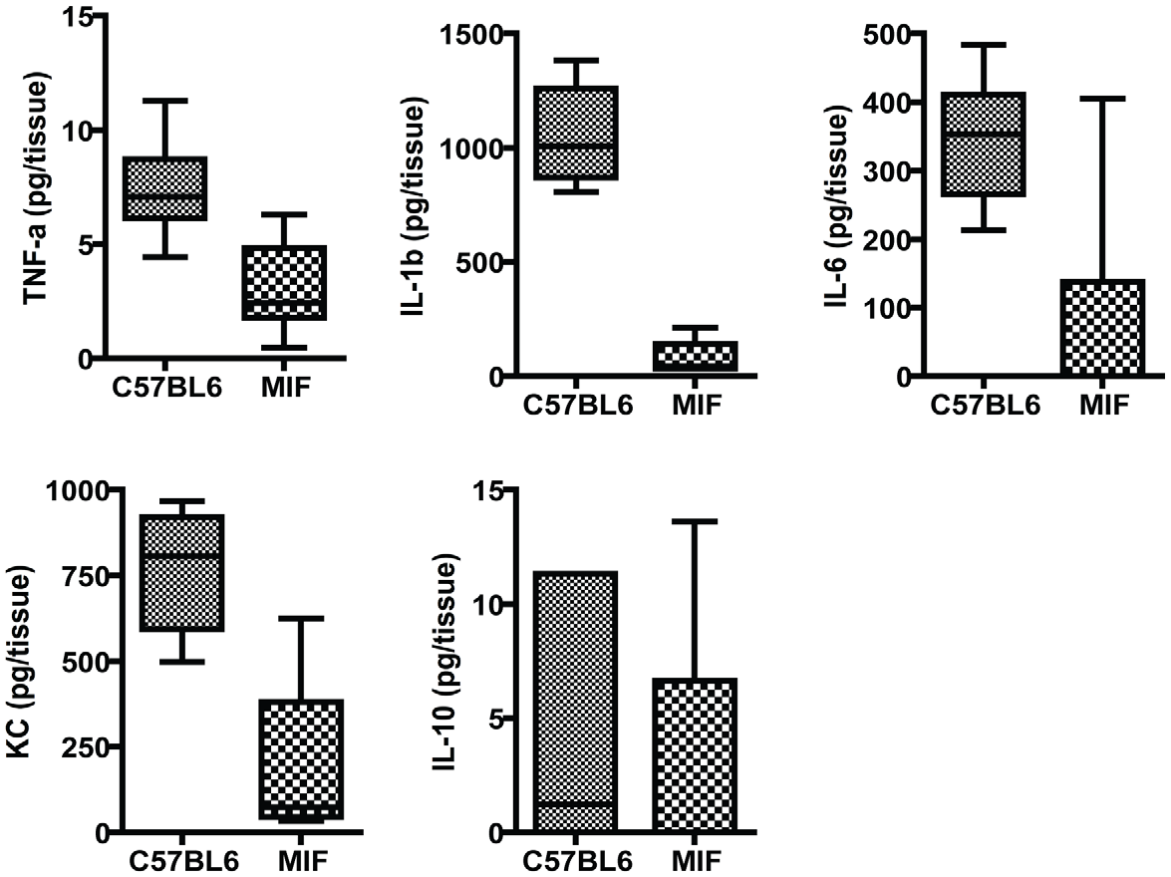
Inflammatory responses in the corneas of MIF KO and C57Bl6 mice infected with *P. aeruginosa* strain 6294. Groups of 7 MIF KO mice and C57Bl6 mice were infected with 1×10^6^ cfu placed onto scratch-injured eyes. Mouse corneas were harvested at 48 h after infection, washed in F12 media, homogenized in PBS containing a mix of protease inhibitors and supplemented with 0.5% Triton to disrupt plasma membranes. The levels of mouse cytokines in corneal lysates were simultaneously measured using a Meso Scale Discovery (MSD) multiplex 7-spot electrochemiluminescence (ECL) assay. The levels of cytokines measured in the different groups were compared with the Mann-Whitney U test. TNF-α, IL-1β, IL-6 and KC were significantly decreased in MIF KO with C57Bl6 mice P = 0.004, P = 0.001, P = 0.01, P = 0.04 respectively.

Since MIF regulates the pro-inflammatory responses to LPS challenge *in vitro* as well as in several *in vivo* models, we examined how MIF might modulate the molecular responses to *P. aeruginosa* induced keratitis by measuring local cytokine production based on prior findings as to which mediators are regulated by MIF and a small-scale microarray analysis we performed (data not shown) on infected corneas from WT and MIF KO mice to suggest which cytokines might be candidates for a more thorough investigation. A panel of five cytokines, TNF-α, IL-1β, KC, IL-6, and IL-10, were quantified at the protein level in infected corneas harvested from individual animals. Infected MIF KO mice had significantly lower levels of TNF-α, IL-1β, IL-6, and KC protein in corneal homogenates compared to corneas from infected C57Bl6 mice. There was no difference in the levels of the anti-inflammatory cytokine IL-10 (Fig. 2).

### MIF deficiency modulates PMN infiltration to the site of infection in the eye

**Figure 3:**
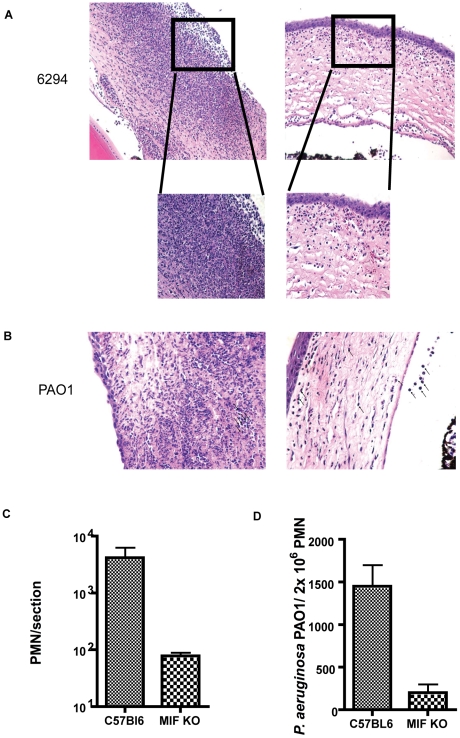
Histological examination of mouse corneas inoculated with *P. aeruginosa* 6294 or PAO1 at 48 h post-challenge. **A.** Groups of C57Bl6 mice (N = 7) and MIF KO (N = 7) were infected with *P. aeruginosa* strain 6294 and the eyes harvested at 48 h post-challenge. Tissue was embedded in paraffin, sectioned and stained with H&E. Representative images were collected at 20× magnification. The inset in the magnified area depicts the morphological changes after 48 h of infection using 40× magnification. **B.** Representative images of H&E tissue sections from C57Bl6 mice and MIF KO infected with *P. aeruginosa* strain PAO1 (20× magnification). **C.** PMN recruitment was quantified by counting PMNs per field of vision in three consecutive sections from 3 individual mice. **D.** Opsonophagocytosis of *P. aeruginosa* strain PAO1 by PMNs from WT C57Bl6 and MIF deficient mice. Bone marrow derived MIF-deficient and MIF-sufficient PMNs were exposed to *P. aeruginosa* PAO1 in the presence of complement and murine sera for 3 h at 37C. Upon completion of incubation PMNs were lysed and bacterial levels in the reaction quantified (p = 0.04, P values determined by an unpaired Student *t*-test).

To determine if differences in inflammatory responses translate into differences in the presence of infiltrating PMNs during infection, histologic observations were carried out. C57Bl/6 mice showed destruction of the epithelial layer of the cornea and elevated infiltration of PMNs in the aqueous chamber. In contrast, in the MIF KO mice, there were less infiltrating PMNs, less edema, and no destruction of the epithelial layer (Fig. 3A and 3B) when either strain 6294 or PAO1 were used to induce infections. The pathology in the C57Bl/6 corneas was consistent with an acute *P. aeruginosa* infection.

The findings on bacterial levels and PMN infiltration into the *P. aeruginosa*-infected cornea indicated that MIF promoted PMN infiltration leading to more pathology while also inhibiting the clearance of this microbe from the cornea. To determine a potential cellular basis for the improved outcomes in MIF-deficient mice, we compared the opsonic killing activity of PMNs obtained from MIF-sufficient and MIF-deficient animals. MIF-deficient PMNs phagocytosed *P. aeruginosa* strain PAO1 more efficiently that MIF-sufficient PMNs, as evident by the decreased number of *P. aeruginosa* recovered from the PMN/bacteria mixture at the end of the experiment (Fig. 3D).

### MIF promotes expression of proinflammatory cytokines in primary corneal epithelial cells

**Figure 4:**
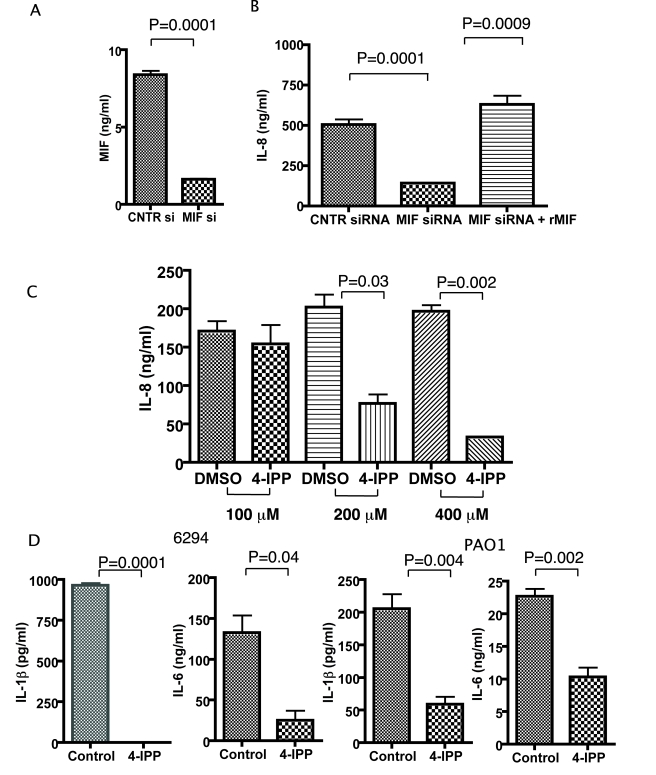
Corneal epithelial cells produce less IL-8, IL-6, IL-1β after infection with *P. aeruginosa* strain 6294 when MIF levels are reduced or MIF tautomerase activity inhibited. **A.** 1×10^6^ corneal epithelial cells were seeded in 6-well plates, transfected with control siRNA or MIF siRNA, and MIF levels in the culture supernatants were measured by ELISA. **B.** 1×10^6^ corneal epithelial cells were infected with *P. aeruginosa* at an MOI 25 for 60 min after 72 h of initial treatment with either control siRNA or MIF siRNA. Recombinant MIF was added to an additional series of wells, pretreated with MIF siRNA. Tissue culture supernatants were collected 6 h post infection and the levels of IL-8 measured by ELISA. The background IL-8 levels were subtracted. Results are representative of duplicate experiments. **C.** Corneal epithelial cells were pre-treated with 100, 200 or 400 µM of 4-IPP prior to infection with *P. aeruginosa* strain 6294. The inhibitor was maintained during the infection and for 6 h post-infection. Supernatants were collected and IL-8 quantified by ELISA. The background levels were subtracted. The results are representative of duplicate experiments. P values determined by an unpaired Student *t*-test. **D.** Corneal epithelial cells were treated with 400 µM of 4-IPP prior to infection with *P. aeruginosa* strain 6294 or PAO1. The inhibitor was maintained during the infection and for 6 h post-infection. Supernatants were collected and IL-1β, IL-6, IL-8 quantified by ELISA. The background levels were subtracted. The results are representative of duplicate experiments. P values determined by an unpaired Student *t*-test.

Since it is well documented that human epithelial cells participate in neutrophil recruitment in response to infection, we determined if MIF affects IL-8 synthesis by corneal epithelial cells [20],[21],[22]. Primary human corneal epithelial cells were grown to confluence, treated with either a siRNA to knock-down MIF or with a control (control) siRNA (Fig. 4A). Seventy-two h after the knock-down cells were infected with *P. aeruginosa* strain 6294 (Fig. 4B). We also evaluated the effect of adding recombinant MIF (rMIF) to cells treated with siRNA for MIF. Tissue culture supernatants were collected and IL-8 levels measured by ELISA. *P. aeruginosa* infection induced IL-8 expression in control siRNA-treated cells, whereas IL-8 production was much less pronounced in MIF siRNA-treated cells (ANOVA, P<0.01) (Fig. 4B). Treatment with rMIF reconstituted *P. aeruginosa*-induced IL-8 production in MIF-siRNA treated cells. Protein concentrations in cellular lysates were determined and no differences found, indicating no major changes in cellular numbers occurred during the experiments.

We next analyzed the effect of a small molecule inhibitor of the MIF tautomerase activity, 4-IPP, on *P. aeruginosa-*induced inflammation. Published studies have demonstrated that 4-IPP forms covalent complexes with MIF, and, hence, inhibits MIF activity [23]. Treatment with 4-IPP almost completely abolished IL-8 synthesis. This effect of 4-IPP was observed at concentrations of 200 µM and 400 µM (Fig. 4C). Since we were concerned that the 4-IPP treatment could induce cellular apoptosis due to the documented role of MIF in promoting cell survival [24],[25], we next analyzed lysates obtained from non-treated and *P. aeruginosa* strain 6294 infected cells in the presence or absence of 400 µM 4-IPP and probed for activated caspase 3 as a marker for apoptosis. At 6 h post infection we did not observe activation of caspase 3, however some activation was detected at 24 h post-infection (Figure S2). Hence, we have performed the majority of our infection experiments within 6 h post-infection. When epithelial cells were stimulated by *P. aeruginosa* strains 6294 or PAO1 both IL-1β and IL-6 secretion was abolished in the presence of the MIF inhibitor (Fig. 4D).

**Figure 5:**
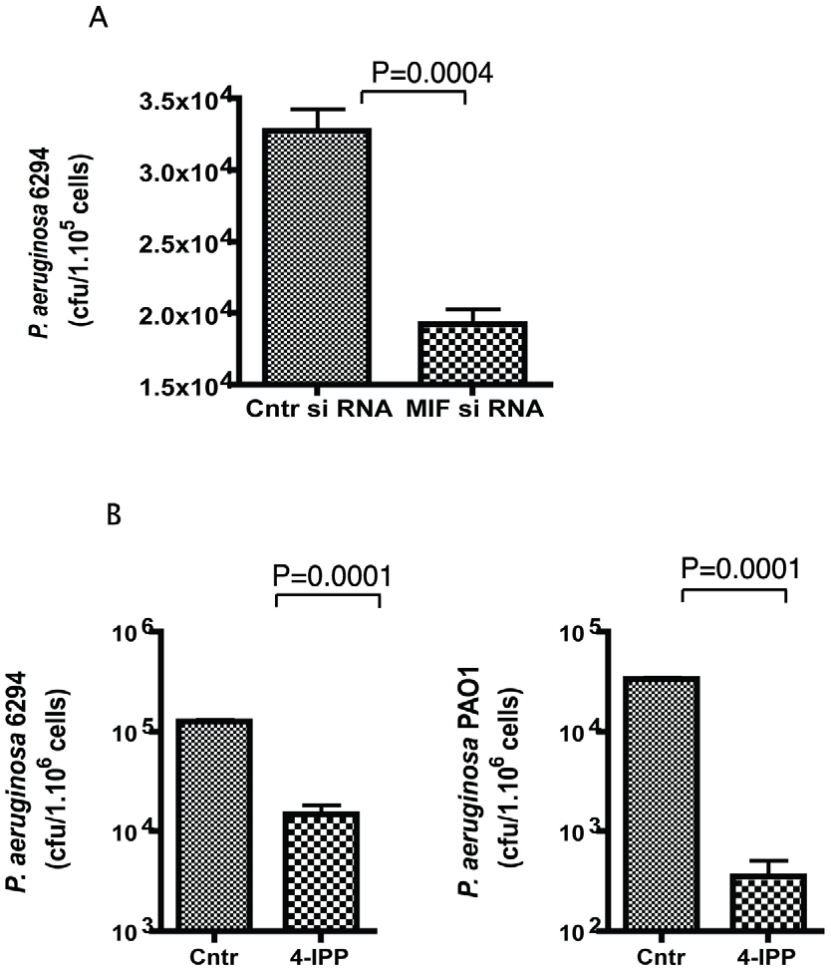
4-IPP inhibits *P. aeruginosa* invasion of corneal epithelial cells. **A.** Corneal epithelial cells were pre-treated with MIF siRNA or control siRNA for 72 h prior to the experiment. Invasion assays were carried out with *P. aeruginosa* strain 6294 at a MOI 10 for 3 h. Cells were washed, treated with gentamicin to kill all extracellular bacteria, lysed and plated. The results are representative of triplicate experiments. P values determined by an unpaired Student *t*-test. **B.** Corneal epithelial cells were pre-treated with 400 µM of 4-IPP prior to infection with *P. aeruginosa* strains 6294 or PAO1 and the inhibitor was maintained during the infection. After 3 h, cells were washed, treated with gentamicin, lysed and plated. The results are derived from triplicate experiments. P values determined by an unpaired Student *t*-test.

As *P. aeruginosa* bacteria found in the cornea during infection are mostly intracellular, we determined if MIF modulated the uptake of bacteria by corneal epithelial cells using *in vitro* invasion assays. Inhibition of MIF by either siRNA treatment or by treatment with small molecule inhibitor 4-IPP, significantly decreased the internalized levels of *P. aeruginosa* in primary human corneal epithelial cells (Fig. 5). These findings suggest that MIF modifies epithelial responses facilitating bacterial entry in the epithelial cells and thus inhibits the niche that *P. aeruginosa* uses to survive and escape from PMNs recruited to the eye in response to infection.

### Recombinant MIF restores the wild-type response to *P. aeruginosa*-induced acute bacterial keratitis in MIF KO mice

**Figure 6:**
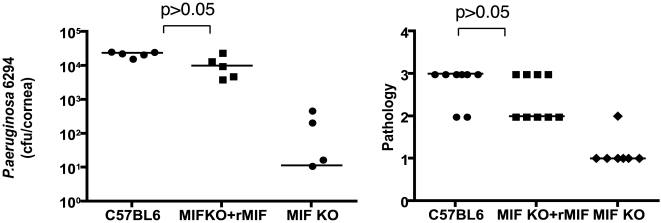
rMIF restores sensitivity to *P. aeruginosa*-induced disease in MIF KO mice. **A.** Topical treatment of corneas from *P. aeruginosa*-infected MIF-deficient mice resulted in bacterial levels comparable to that achieved in infected WT C57Bl6 mice (P>0.05 ANOVA and Dunnett's test) while MIF KO mice had reduced levels of *P. aeruginosa* in the tissue. **B.** Pathology scores. MIF KO presented with reduced pathology scores (P<0.05, ANOVA and Dunnett's test). Points indicate values from individual mice, bars the medians. The data are from one representative experiment out of two performed.

To verify that MIF was the major factor responsible for the reduced infectious complications of *P. aeruginosa* keratitis in MIF KO mice, reconstitution experiments were performed. One µg of rMIF administered topically onto the eye of infected MIF KO mice restored the wild-type response to infection with *P. aeruginosa* strain 6294 (Fig. 6). This increased bacterial burden in the reconstituted MIF KO correlated with elevated pathology scores, indicative of increased susceptibility to infection.

### Inhibition of MIF activity by 4-IPP promotes recovery from *P. aeruginosa*-induced keratitis

**Figure 7:**
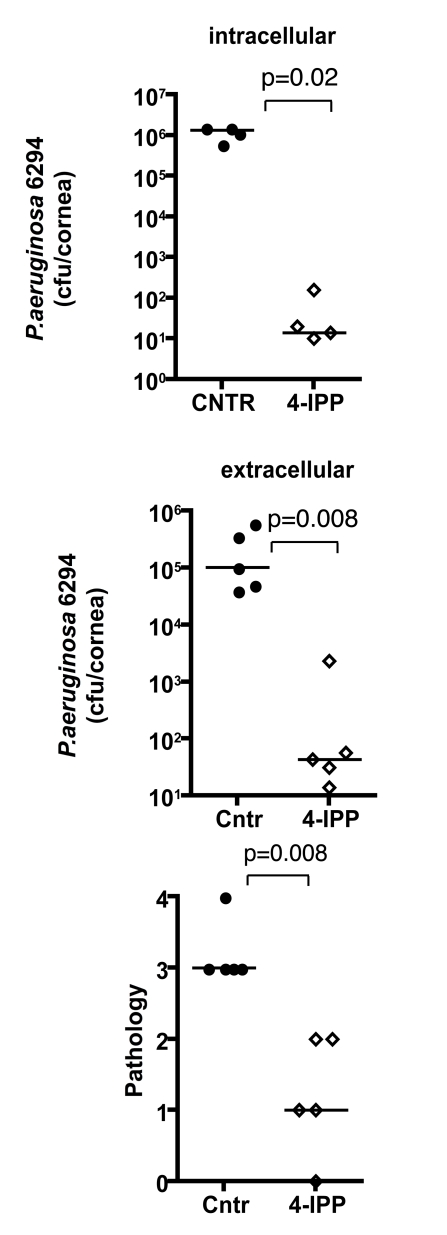
Treatment with 4-IPP promotes recovery from *P. aeruginosa*-induced corneal infection and pathology. Groups of C57Bl6 mice were treated with either gentamicin alone or gentamicin and 4-IPP for 5 days after infection after which infected corneas were harvested, homogenized, and bacteria quantified. Points indicate values from individual mice, bars the medians. The data are from one representative experiment out of two performed.

To analyze the effect of MIF inhibition during *P. aeruginosa*-induced keratitis, cohorts of C57Bl6 mice were infected with 5×10^5^ cfu/mouse of *P. aeruginosa* strain 6294 and treated topically with gentamicin starting 24 h post-infection. A separate cohort of infected C57Bl6 mice received both 4-IPP IP and gentamicin eye-drops on the same schedule. Animals were monitored for 5 days to analyze recovery from infection and the infected corneas were harvested to quantify the intracellular and extracellular bacteria. Mice that received the 4-IPP and gentamicin treatment cleared *P. aeruginosa* more efficiently than did the group treated only with gentamicin as demonstrated by the significantly decreased extracellular and intracellular cfu recovered from the corneas, and decreased pathology scores (Fig. 7).

## Discussion

*Pseudomonas aeruginosa* causes serious corneal disease, associated with acute ocular inflammation. Both bacterial and host factors contribute to the pathogenesis of *P. aeruginosa* keratitis, indicating that the optimal interventions for this infection will involve both control of bacterial levels and modulation of inflammation to limit pathology while not compromising bacterial clearance. Here, we demonstrated that a master regulator of inflammation–MIF–promoted both pathology associated with *P. aeruginosa* corneal infection and inhibited bacterial clearance as shown by outcomes in MIF-KO mice which had decreased bacterial burdens in the corneas, decreased inflammation, and decreased neutrophil infiltration compared to MIF-sufficient WT mice. These improved outcomes in MIF-deficient mice could be reversed by treating *P. aeruginosa*-infected eyes with rMIF and could be mimicked in WT mice treated with the MIF inhibitor 4-IPP. Thus, it appears that one host molecule makes a major contribution to both aspects of disease resulting from *P. aeruginosa* corneal infection, indicating a high potential for ameliorating the consequences of this infection by therapies that target the activities of MIF.

To define mechanisms associated with the effects of MIF in *P. aeruginosa* keratitis we used in vitro studies to show that reduction of MIF levels by siRNA treatment or by the use of the tautomerase inhibitor of MIF, 4-IPP, results in decreased production of IL-8, IL-1β, and IL-6 by human corneal epithelial cells after infection. Consistent with this finding, the absence of MIF *in vivo* also reduced inflammatory responses to *P. aeruginosa* during experimental murine corneal infection. Furthermore, we also found that PMNs from MIF-deficient mice had a better innate ability to kill *P. aeruginosa* in an *in vitro* opsonic-killing assay, indicating a likely cellular basis for the better control of infection in thee animals. Of note, Niederkorn and colleagues [18] found that MIF inhibits perforin release by NK cells and inhibits NK cell motility, indicating a contribution of MIF to the immune-privileged status of the eye. Thus, it appears in the basal state MIF limits cellular activation, but a consequence of this is that the responses to infection are dampened and the increased infiltration of PMN into the infected cornea that is needed to deal with the increased bacterial burdens results in more tissue pathology. A similar improved outcome from *P. aeruginosa* lung infection was reported for MIF-deficient mice [11].

Successful recovery form acute bacterial infection depends on timely recruitment of neutrophils. PMNs are present in the cornea and aqueous chamber in MIF deficient mice at reduced levels when compared to infected WT control mice, yet, there are sufficient numbers of PMNs recruited in the MIF KO to clear bacteria, as demonstrated by the decreased levels of *P. aeruginosa* measured at 48 h after the onset of infection. Hence, MIF deficiency both reduces pathology during infection and still allows for sufficient host responses to clear bacteria. Our data suggests that in infected mouse corneas, inflammatory mediators such as KC or MIP2 are initially released by corneal epithelial cells or keratocytes which in turn recruit PMNs to effectively handle bacterial infections in the eye [26], a process that occurs in the infected MIF KO mice leading to resolution of infection. In contrast, in the infected C57Bl6 mice these mediators are produced at higher levels and become part of a classic hypersensitivity response that contributes to tissue damage and elevated pathology [27],[28]. Taken together these data suggest that by modulating the level of inflammation occurring in the eye triggered by *P. aeruginosa* infection it is possible to promote expression of proinflammatory mediators sufficient to control infection while also preventing inflammation-induced corneal pathology and associated loss of vision.

## Materials and Methods

### Mice

Ethics Statement: All studies were performed in accordance with the Harvard Medical School Institutional Animal Care and Use Committee guidelines (approval date 10/14/2008; number IACUC 02432).

Breeding pairs of *mif* knock-out (KO) mice were a kind gift from Dr. C. Gerard (Children's Hospital Boston). Control mice (C57Bl6) were obtained from Taconic Farms. Mice were housed and bred in the Channing Laboratory Animal Care Facilities. Mice were housed and bred in one of the Harvard Medical Area Animal Care Facilities.

### Bacterial strains and inocula

*P. aeruginosa* strains PAO1 and 6294 were used throughout these experiments. Effects from low dose (5×10^5^ cfu/eye) and high dose (1×10^7^ cfu/eye) inocula were evaluated to determine the doses to use in the *in vivo* infection studies. Generally, the bacterial strains were grown o/n at 37C on Tryptic Soy Broth agar plates prior to experiments.

### Infection model

The experimental protocols were approved by the Institutional Animal Care and Use Committee of the Harvard Medical Area Office for Research Subject Protection and were consistent with the Association for Research in Vision and Ophthalmology guidelines for studies in animals. Infections were carried out as described previously [29]. Mice were anesthetized with ketamine and xylazine injections. Three 0.5 cm scratches were made on the cornea and an inoculum of *P. aeruginosa* delivered in 5 µl onto the eye. Mice remained sedated for about 30 min. For evaluation of corneal pathology, daily scores are recorded by an observer unaware of the experimental status of the animals based on a the following scoring system using a graded scale of 0 to 4 as follows: 0, eye macroscopically identical to the uninfected contra-lateral control eye; 1, faint opacity partially covering the pupil; 2, dense opacity covering the pupil; 3, dense opacity covering the entire anterior segment; and 4, perforation of the cornea, phthisis bulbi (shrinkage of the globe after inflammatory disease), or both. To determine the levels of bacteria in the cornea 24 or 48 h after infection, mice were sacrificed, eyes enucleated and corneas dissected from the ocular surface. To quantify the extracellular levels of *P. aeruginosa*, corneas were excised and suspended in PBS, vortexed, serial dilutions made and plated on *P. aeruginosa* selective cetrimide plates. To measure the intracellular levels of *P. aeruginosa*, the intact corneas were placed in F12 medium containing 5% FBS and 300 µg gentamicin/ml for 60 min, vigorously rinsed to wash away the antibiotic and the tissue then homogenized in 0.05% Triton X100 in 5% FBS-F12, diluted in 5% FBS-F12, and plated for bacterial counts. For treatment, groups of mice were infected with *P. aeruginosa* strain 6294, treated topically daily for 5 days with 5 µl of a solution of 100 µg gentamicin/ml, along with 50 µl of 1 M 4-IPP injected IP. The 4-IPP was solubilized in DMSO/corn oil [23]. Bacteria were quantified 5 days after infection by homogenizing and plating the corneal tissue.

### Reconstitution experiments

Mice were infected with *P. aeruginosa* strain 6294 and 1 µg recombinant MIF (rMIF). applied topically in 5 µl at the time of infection and subsequently every 6 h after the infection. Corneas were harvested after 48 h of infection, treated with 300 µg gentamicin/ml for 60 min, washed, homogenized with F12 medium containing 5% FBS and 0.05% Triton X100, diluted and plated.

### Histopathology examinations

Eyes were enucleated from euthanized mice and fixed in 4% paraformaldehyde then embedded in paraffin. Four µm sections were cut, and stained with hematoxylin-eosin to visualize tissue morphology following previously used techniques [30].

### *In vitro* infection assays

Primary corneal epithelial cells [31] were grown in 6-well plates in either keratinocyte SFM (Invitrogen) medium supplemented with antibiotics, EGF, and pituitary gland extract or in MEM/F12 mix supplemented with EGF, insulin, DMSO, and cholera toxin [31]. Confluent monolayers of corneal cells were treated for 1 h with 4-IPP then infected with different strains of *P. aeruginosa* (PAO1, 6294) at a MOI of 25 for 1 h. Following treatment for 1 h with 300 µg gentamicin/ml, cells were re-supplemented with growth medium. The keratinocyte SFM growth medium was supplemented with penicillin, streptomycin, EGF, and pituitary gland extract. 6-iodo-6-phenylpirimidine (4-IPP) was used to inhibit MIF activity *in vitro* and was maintained during the infection and gentamicin treatments. Different concentrations of 4–IPP were used, ranging from 100 µM to 400 µM. The tissue culture supernatants were collected at 6 h and 24 h after infection and total cell lysates were prepared in RIPA buffer (50 mM Tris-HCl, pH 7.5, 150 mM NaCl, 1% Triton X-100, 1% sodium deoxycholate, 1% SDS, and Complete Mini Protease Inhibitor Cocktail (Roche Diagnostics).

### Epithelial cell invasion assay

*P. aeruginosa* strains 6294 or PAO1 were grown to mid-log phase in TSB for 2 h at 37C and used for invasion assays. Bacterial cells were washed and suspended in F12 medium. Human primary corneal epithelial cells were grown to confluence in 6 well plates in F12 medium and infected with the *P. aeruginosa* strains at a MOI of 25 for 3 h. After the infection, cells were washed with F12, then treated with 300 µg or 400 µg gentamicin/ml for 1 h to kill the extracellular bacteria depending on the activity of the antibiotic. After washing away the antibiotic, cells were lysed in 1% Triton X 100 in MEM and the lysates were diluted then plated on cetrimide plates to enumerate intracellular bacteria.

### Cytokine analysis

Commercially available ELISA assays (R&D Systems) were used to determine the levels of cytokines (IL-8, IL-6) produced by *P. aeruginosa* infected primary human corneal cells. Mouse cytokines were measured using a Meso Scale Discovery (MSD) multiplex 7-spot electrochemiluminescence (ECL) assay and outputs measured by an ultra low noise charge-coupled device (CCD) Imager 2400 (Meso Scale Discovery, Gaithersburg, MD, USA). The cytokines measured included interleukin (IL)-1β, IL-6, IL-12p70, IL-10, IFNγ and the alpha chemokine neutrophil attractant and activator CXCL1/GRO (also known as KC). The MSD ECL platform has been previously validated against cytokine standards recommended by WHO and U.K. National Institute for Biological Standards and Control (NIBSC) and by comparison to traditional ELISA [32].

### siRNA experiments

Production of MIF by the primary corneal epithelial cells was reduced by MIF-specific siRNA treatment. Briefly, cells were transfected with MIF-specific or control siRNA (On-target plus SMART pool; Dharmacon) by incubating with a transfection mixture that consists of 50 nM siRNA (Dharmacon) and 6 µl Oligofectamine (Invitrogen) in 1 ml of Opti-MEM (Gibco) for 48 h, after which the cells were maintained with regular growth medium. Epithelial cells were infected with *P. aeruginosa* (strain 6294 or strain PAOI) 48 h to 72 h after siRNA treatment. A non-siRNA treated control was also included to determine if there is an effect on inflammatory gene expressing from the control siRNA.

### Purification of PMNs and opsonophagocytic assays

Murine PMNs were purified from bone marrow by flushing the cells out of the tibias and femurs with 3 ml HBSS (Hanks balanced salt solution) buffer (Invitrogen). Cells were pelleted by centrifugation at 380 g for 10 min at tabletop centrifuge; resuspended in 1 ml HBSS and overlayed on the top of a Histopaque gradient composed of 3 ml Histopaque 1119 (Sigma) and 3 ml Histopaque 1077 (Sigma). Cells were centrigufed for 30 min at 700 g, room temperature, without a brake. Neutrophils were collected from the interface of the Histopague 1077 and 1119, transferred to a fresh tube and washed three times by adding 10 ml og HBSS. Viable cells were enumerated by tryptan blue exclusion technique.

Opsonophagocytic assays were performed by mixing 100 µl of 2.5×10^6^ PMN with 100 µl of 400 fold dilution of *P. aeruginosa* grown in TSB to reach mid-log phase as estimated by OD _650_ reading of 0.4. 100 µl of Rabbit complement sera (Sigma) and 100 µl of freshly harvested mouse sera from either C57Bl6 or MIF KO mice were added to the sample mixture as appropriate. The reaction was incubated for 90 min at 37°C with rotation. After completion of the incubation an aliquot was taken and lyzed with 0.05% Tween 20/TSB. Bacteria were enumerated by plating on *P. aeruginosa* selective cetrimide plates.

## Supporting Information

Figure S1 MIF KO mice are protected from *P. aeruginosa* PAO1-induced keratitis 48 h after infection. Extracellular and intracellular levels of *P. aeruginosa* strain PAO1 48 h following infection with 10^6^ cfu onto the eyes of WT C57/Bl6 or MIF KO mice. Points indicate individual mice, bars the medians. Significantly different levels of PAO1 were detected both extracellularly P = 0.03 and intracellularly P = 0.0025, Mann-Whitney U test. Pathology scores. P = 0.008, Mann-Whitney U test. The data are from one representative experiment out of three performed.
(1.25 MB EPS)

Figure S2 Cellular apoptosis during infection and 4-IPP treatment. A. Corneal epithelial cells were infected with either *P. aeruginosa* 6294 in the presence of 4-IPP or DMSO control treatment and cellular lysates were collected at either 6 h post-infection or 24 h post-infection. Lysates were loaded on 4–12% SDS-PAGE gel, blotted and developed with antibody to activated caspase-3 or antibody to actin. B. Corneal epithelial cells were infected with either *P. aeruginosa* 6294 in the presence of 4-IPP or DMSO control treatment, 6 h post-infection cells were trypsinized and live/dead bacteria were quantified by Tryptan Blue exclusion assay. The percent of live cells were plotted.
(0.66 MB EPS)
